# A Phase 4, multicenter, prospective, non-interventional, observational study to investigate the effectiveness and safety/tolerability of perampanel when used as first adjunctive therapy in routine clinical practice in people with epilepsy: Study 512

**DOI:** 10.3389/fneur.2025.1533767

**Published:** 2025-04-15

**Authors:** Sergey Burd, Giovanni Assenza, Sofia Quintas, Francisco José Gil López, Jan Wagner, Anna Lebedeva, Pavel Vlasov, Nina Pantina, Anna Patten, Samantha Goldman, Ricardo Sáinz-Fuertes, Marta Torres Arlandis, Stanislas Lagarde, Tobias Sejbaek, Vadim Kharkovsky

**Affiliations:** ^1^Pirogov Russian National Research Medical University, Moscow, Russia; ^2^Federal Center of Brain Research and Neurotechnologies, Moscow, Russia; ^3^UOC Neurologia, Fondazione Policlinico Universitario Campus Bio-Medico, Rome, Italy; ^4^Research Unit of Neurology, Department of Medicine and Surgery, Università Campus Bio-Medico di Roma, Rome, Italy; ^5^Centro Hospitalar Universitário Lisboa Norte, Lisbon, Portugal; ^6^Hospital Universitari del Sagrat Cor, Barcelona, Spain; ^7^University of Ulm and Universitäts-und RehabilitationsklinikenUlm, Ulm, Germany; ^8^Department of Neurology, Scientific Research Institute of Clinical Medicine named after N.A. Semashko, Russian University of Medicine, Moscow, Russia; ^9^Eisai Europe Ltd., Hatfield, Hertfordshire, United Kingdom; ^10^APHM, Timone Hospital, Epileptology and Cerebral Rhythmology, Marseille, France; ^11^Aix Marseille Univ, INSERM, INS, Inst Neurosci Syst, Marseille, France; ^12^Hospital Southwest Jutland, University Hospital of Southern Denmark, Esbjerg, Denmark; ^13^University of Southern Denmark, Odense, Denmark

**Keywords:** epilepsy, first add-on, focal epilepsy, generalized epilepsy, perampanel, real-world

## Abstract

**Introduction:**

Study 512 aimed to assess the efficacy and safety of perampanel (PER) as the first add-on therapy.

**Methods:**

In this 12-month, prospective, observational, multicenter study, people with epilepsy (PWE) aged ≥12 years with focal-onset seizures or generalized tonic–clonic seizures (GTCS) associated with idiopathic generalized epilepsy received PER as the first add-on therapy to antiseizure medication (ASM) monotherapy. The primary efficacy endpoint was the retention rate at 12 months. Other endpoints included change in seizure frequency from baseline; pragmatic seizure freedom rate (proportion of PWE in the full analysis set achieving freedom from all seizures); responder rate (≥50% seizure frequency reduction from baseline), changes from baseline in the 10-item Quality of Life in Epilepsy questionnaire (QOLIE-10) total score, the Epworth Sleepiness Scale (ESS), and the age-corrected EpiTrack and EpiTrack Junior total score; safety/tolerability (treatment-emergent adverse events [TEAEs]); and PER dose.

**Results:**

Of 184 PWE (Safety Set, *n* = 182; Full Analysis Set, *n* = 174), 135 (73.4%) completed the 12-month study. The mean PER dose was 4.7 mg/day. Retention rate at 12 months was 74.2% in the overall population, 81.8% in the 12 to <18 years age group, 74.3% in the 18 to <65 years age group, and 66.7% in the ≥65 years age group. Retention rates were similar between PWE with focal-onset seizures (74.5%) and GTCS (75.0%). The median reduction in monthly seizure frequency per 28 days from baseline to 12 months was 78.6% in the overall population, 92.3% in the 12 to <18 age group, 75.0% in the 18 to <65 years age group, and 87.5% in the ≥65 years age group. In the overall population, pragmatic seizure freedom rates at 12 months were 36.2% (all seizures), 34.1% (all focal seizures), and 45.5% (GTCS); the responder rate at 12 months was 64.4% in the overall population. In total, 52.7% of PWE experienced TEAEs, and 12.1% discontinued due to TEAEs. No significant changes were identified from baseline to 12 months in QOLIE-10, ESS, and the age-corrected EpiTrack and EpiTrack Junior scores.

**Conclusion:**

PER was efficacious for focal and generalized seizures across all age groups and was generally well-tolerated, as demonstrated by the high retention rates at 12 months.

## 1 Introduction

Approximately half of people with epilepsy (PWE) fail to achieve seizure control with the first prescribed antiseizure medication (ASM) (1). When monotherapy fails, adjunctive therapy is often initiated to improve seizure control (2), with a significant proportion of PWE achieving seizure freedom with a second or third concomitant ASM (28 and 24%, respectively) (3). However, the probability of achieving seizure control diminishes with each subsequent regimen (3). As treatment with multiple ASMs could be associated with negative side effects, there is a need for ASMs that are well-tolerated and effective as first adjunctive therapy (3).

Perampanel (PER) is a first-in-class, non-competitive antagonist of the ionotropic *α*-amino-3-hydroxy-5-methyl-4-isoxazolepropionic acid (AMPA) glutamate receptor on post-synaptic neurons (4). PER is approved in over 75 countries, including the United States (US), European Union (EU), and Russia, as monotherapy or adjunctive therapy for the treatment of both focal-onset seizures, with or without focal to bilateral tonic–clonic seizures (FBTCS), and generalized tonic–clonic seizures (GTCS) (5–7).

Approval of PER for the treatment of focal-onset seizures was based on the results of three Phase III randomized, double-blind, placebo-controlled trials (8–10), and its approval for the treatment of GTCS in idiopathic generalized epilepsy (IGE) was based on the results of one Phase III, randomized, double-blind, placebo-controlled trial (11). However, all these trials included PWE who had previously failed treatment with at least two prior ASMs and/or were still experiencing seizures despite currently taking stable doses of one to three ASMs before the initiation of PER.

Following PER’s approval, a few observational studies indicated that PER is efficacious as early add-on therapy in those individuals who do not respond to ASM monotherapy (12, 13). Additionally, a *post-hoc* analysis from the PERMIT study (a pooled analysis of real-world studies of PER) demonstrated that PER was significantly more effective and better tolerated in PWE who initiated PER as an early add-on therapy than in PWE who received PER as a later add-on therapy (14). An Italian consensus clinical practice statement acknowledged that PER has many features that favor its use as a first add-on therapy (15).

However, *prospective* evidence for the use of PER as a first add-on therapy is currently limited. Here, we report the results of Study 512, a prospective, observational study evaluating the use of PER in routine clinical practice as the first add-on therapy to baseline ASM monotherapy in PWE aged ≥12 years with focal-onset seizures and GTCS associated with IGE.

## 2 Materials and methods

### 2.1 Study design

**Figure 1:**
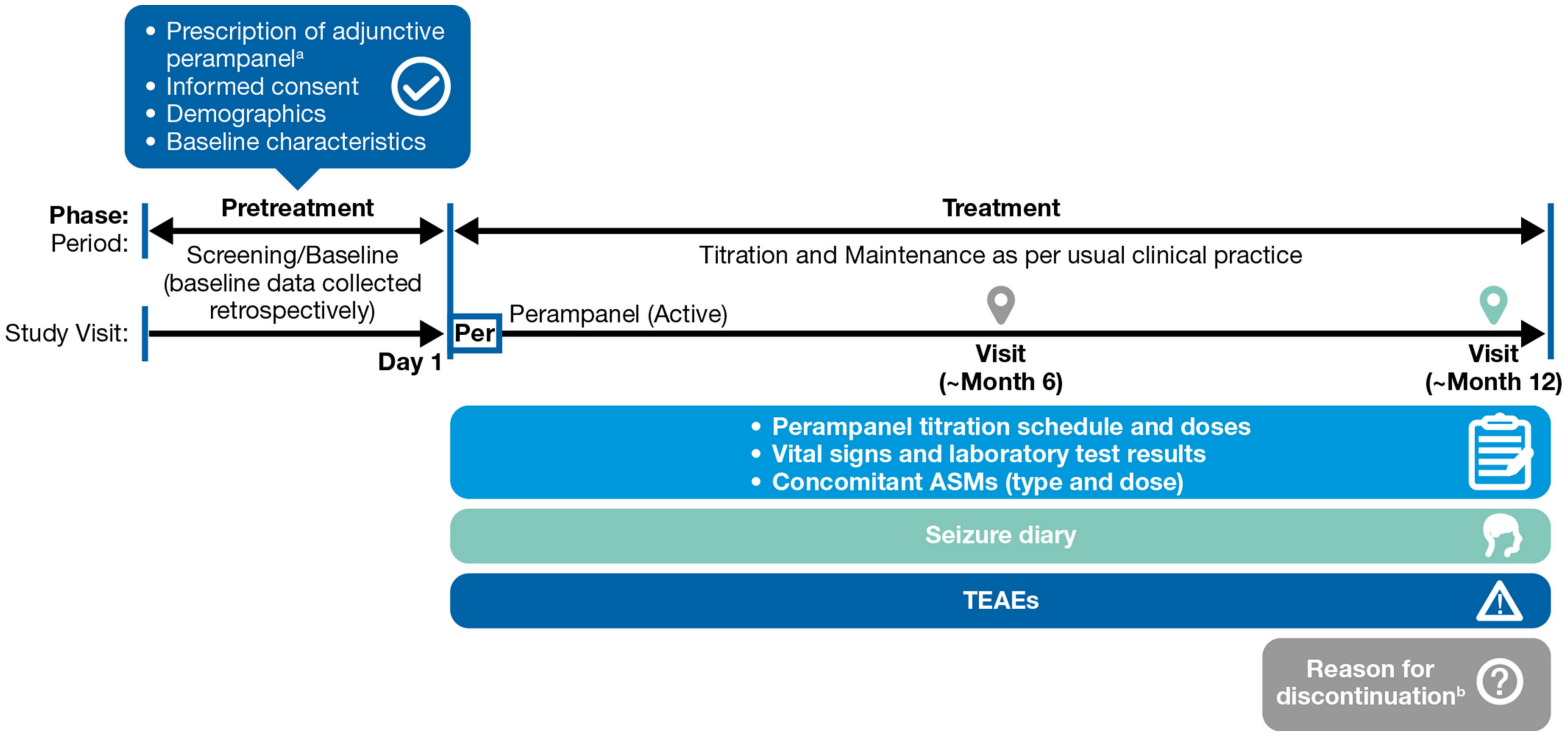
Study design. ^a^Based on the treating physician’s clinical judgment, patients who had seizures while receiving one other ASM as monotherapy. The decision to prescribe perampanel was made before and independently of the physician’s decision to include the patient in the study. ^b^If the patient withdrew from the study or discontinued perampanel treatment, the assessments of the final visit were collected (if part of routine clinical practice), and the reason for early termination was recorded. ASM, antiseizure medication, PER, perampanel; TEAE, treatment-emergent adverse event.

Study 512 was a 12-month prospective, observational, multicenter study conducted across seven countries (selected because of their high rate of PER use as early add-on therapy): Denmark, France, Germany, Italy, Portugal, Russia, and Spain (ClinicalTrials.gov identifier: NCT04252846). Patients were assessed at baseline and as per routine clinical care, along with study visits at approximately 6 and 12 months post-baseline. Final visit assessments were collected in case of PER discontinuation or withdrawal from the study (if available and part of routine clinical practice) (Figure 1). Data were obtained from medical records, seizure diaries, and healthcare providers’ interviews with patients/caregivers at clinic visits.

### 2.2 Ethical statement

The protocol, informed consent form, and appropriate related documents were submitted to Independent Ethics Committees (IECs) for review. The study was initiated only after receipt of documented IEC approval of the protocol and informed consent form. All protocol amendments were reviewed and approved by the IEC before implementation. This study was conducted in accordance with the Principles of the World Medical Association Declaration of Helsinki 2013; International Council for Harmonisation of Technical Requirements for Pharmaceuticals for Human Use (ICH) E6 Guideline for Good Clinical Practice of the European Agency for the Evaluation of Medicinal Products, Committee for Proprietary Medicinal Products (CPMP/ICH/135/95); and European Good Clinical Practice Directive 2005/28/EC and Clinical Trial Directive 2001/20/EC for studies conducted within any EU country. As required, all suspected unexpected serious adverse reactions were reported to the Competent Authorities of all involved EU member states. Written informed consent from each participant in this study was collected before the screening procedure.

### 2.3 Study population

PWE aged ≥12 years were eligible for participation if they had a diagnosis of epilepsy and a history of focal-onset seizures with or without FBTCS or GTCS associated with IGE [according to the International League Against Epilepsy [ILAE] Classification of Epileptic Seizures, 1981 and ILAE Classification of Epileptic Syndromes, 1989; following recruitment, the terminology was then updated in line with the ILAE 2017 classification (16–18)]. PWE needed to have documented focal-onset seizures or GTCS associated with IGE within the past 12 months and seizure diary data for the previous 4 weeks before study initiation, or sufficient clinical details to calculate baseline seizure frequency, and to have been previously treated with one or two ASMs as monotherapy. Key exclusion criteria included episode(s) of status epilepticus within the past 6 months before screening, previous treatment with two or more ASMS in combination (other than during cross-titration between ASM monotherapies), previous or current use of PER, and sensitivity to PER. Participants were prescribed PER as the first add-on therapy to ASM monotherapy based on the treating clinician’s recommendation as per PER’s indication.

### 2.4 Study assessments

The primary efficacy endpoint was the retention rate at 12 months of PER treatment. Secondary efficacy endpoints included retention rate at 6 months; change in seizure frequency from baseline to 6 and 12 months; pragmatic and completer seizure freedom rates at 6 and 12 months; responder rate at 6 and 12 months; and seizure worsening rate at 6 and 12 months. The seizure freedom rate was defined as the proportion of PWE free of all seizures at 6 months (and the previous 3 months) and at 12 months (and the previous 6 months). Pragmatic seizure freedom rates were calculated in the Full Analysis Set (FAS) and completer seizure freedom rates in individuals on PER at that time point. The responder rate was defined as the proportion of PWE in the FAS having at least a 50% reduction in seizure frequency compared to baseline. The sensitivity analysis was based on the subset of the Safety Analysis Set (SAS) with seizure or investigator opinion data. Information on PER dosing (duration, speed of titration, and final dose) was also collected.

Changes from baseline to 6 and 12 months in the 10-item Quality of Life in Epilepsy questionnaire (QOLIE-10) total score (in adult PWE [aged ≥18 years] only) (19), the Epworth Sleepiness Scale (ESS; adult PWE only) (20), and the age-corrected EpiTrack and EpiTrack Junior total score (21, 22) were assessed as exploratory endpoints, along with changes in concomitant ASMs (started before or after PER initiation).

The safety and tolerability of PER were evaluated throughout the study by evaluating the incidence, type, and severity of treatment-emergent adverse events (TEAEs), TEAEs leading to discontinuation, and vital signs.

### 2.5 Statistical analysis

Safety analyses were performed for the SAS population (all PWE who received at least one dose of PER and had at least one post-dose safety measurement), while effectiveness analyses were performed in the FAS population (all PWE who received at least one dose of PER and had at least one post-dose measurement of seizure outcomes).

Descriptive statistics were used for quantitative and qualitative assessments. Quantitative variables were summarized as mean, standard deviation (SD), median, minimum, maximum, and 95% confidence intervals (CIs). Kaplan–Meier methodology was used for retention. Univariate and multivariate logistic regression analyses were used to identify predictors of PER retention and seizure freedom (pragmatic seizure freedom analysis only). Last observation carried forward (LOCF) analyses were applied for responder rates and exploratory endpoints. Statistical analyses were performed using Statistical Analysis System version 9.3 or later or other validated statistical software as required.

## 3 Results

### 3.1 Study population

**Figure 2:**
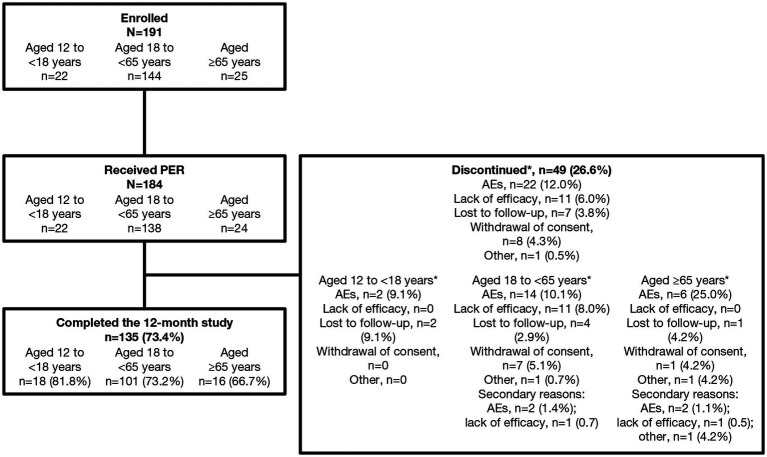
Patient disposition. *Primary reason for discontinuation. AE, adverse event; PER perampanel.

Of the 191 patients enrolled in the study, 184 received PER, of whom 182 were included in the SAS and 174 in the FAS. In total, 73.4% (135/184) completed the 12-month study and 26.6% (49/184) discontinued; the main reasons for discontinuation were AEs (12.0%) and lack of efficacy (6.0%) (Figure 2). The most common (more than one individual) TEAEs leading to discontinuation were irritability (2.2%, *n* = 4), aggression, dizziness, somnolence, seizures, and vertigo (all 1.1%, *n* = 2).

Overall, 51.6% of PWE were female, and the mean (SD) age was 39.3 (19.3) years, with the majority (74.7%; 136/182) aged 18 to <65 years (Table 1). The overall proportion of PWE with psychiatric comorbidities was 18.1% (33/182), which was highest in the 12 to <18 years age group (27.3%; 6/22). The mean (SD) duration of epilepsy was 7.9 (10.2) years, with the majority of PWE having focal-onset seizures (79.1%; 144/182) and 23.6% (43/182) having generalized-onset seizures (Table 2). The most common epilepsy etiologies were structural brain anomalies or malformations (15.4%), genetic (10.4%), stroke (6.0%), and head injury/cranial trauma (5.5%); however, the etiology was unknown for approximately half of the PWE. There were differences in the epilepsy characteristics between the different age groups. The proportion of PWE with focal-onset seizures increased with age. In contrast, the proportion of those with generalized-onset seizures decreased with age, and both epilepsy syndromes (GTCS only, juvenile myoclonic epilepsy [JME], and juvenile absence epilepsy) and genetic etiology were reported more frequently in the 12 to <18 age group than in the ≥65 age group.

**Table 1:** Demographic characteristics at baseline (SAS).

	Age group			Total *N* = 182
Characteristic	12 to < 18 years *n* = 22	18 to < 65 years *n* = 136	≥65 years *n* = 24	
Age, years				
*n* ^a^	22	136	24	182
Mean (SD)	15.0 (1.7)	37.2 (13.7)	73.2 (6.7)	39.3 (19.3)
Median (range)	16 (12–17)	35 (18–64)	71 (65–84)	36 (12–84)
Sex, *n* (%)				
*n* ^a^	22	136	24	182
Female	10 (45.5)	77 (56.6)	7 (29.2)	94 (51.6)
Male	12 (54.5)	59 (43.4)	17 (70.8)	88 (48.4)
Race, *n* (%)				
*n* ^a^	20	123	22	165
White	20 (100)	121 (98.4)	22 (100)	163 (98.8)
Black or African American	0	1 (0.8)	0	1 (0.6)
Asian	0	1 (0.8)	0	1 (0.6)
American Indian or Alaskan Native	0	0	0	0
Native Hawaiian or other Pacific Islander	0	0	0	0
Other	0	0	0	0
Ethnicity, *n* (%)				
*n* ^a^	20	123	22	165
Hispanic or Latino	1 (5.0)	18 (14.6)	6 (27.3)	25 (15.2)
Not Hispanic or Latino	19 (95.0)	105 (85.4)	16 (72.7)	140 (84.8)
Weight, kg				
*n* ^a^	18	113	21	152
Mean (SD)	62.6 (18.2)	71.0 (14.3)	73.0 (8.4)	70.3 (14.3)
Median (range)	59 (33–93)	69 (41–111)	73 (60–85)	69 (33–111)
Height, cm				
*n* ^a^	16	112	21	149
Mean (SD)	161.8 (9.2)	171.0 (10.0)	169.7 (8.8)	169.8 (10.1)
Median (range)	160 (149–177)	170 (150–198)	171 (150–182)	170 (149–198)

^aNumber of PWE for whom datum was available; ASM, antiseizure medication; PWE, people with epilepsy; SAS, Safety Analysis Set; SD, standard deviation.^

**Table 2:** Epilepsy characteristics at baseline (SAS).

	Age group			Total *N* = 182
Characteristic	12 to < 18 years *n* = 22	18 to < 65 years *n* = 136	≥65 years *n* = 24	
Age at epilepsy diagnosis, years				
*n* ^a^	22	134	24	180
Mean (SD)	13.0 (3.0)	28.7 (15.9)	65.0 (13.6)	31.6 (20.3)
Median (range)	13.0 (4–17)	24.0 (1–62)	67.5 (31–83)	24 (1–83)
Duration of epilepsy, years				
*n* ^a^	22	134	24	180
Mean (SD)	2.0 (2.9)	8.8 (10.4)	8.1 (11.2)	7.9 (10.2)
Median (range)	0.5 (0–11)	5.0 (0–52)	3.5 (0–45)	3.0 (0–52)
Seizure type^b^, *n* (%)				
*n* ^a^	22	136	24	182
Focal-onset seizures	12 (54.5)	108 (79.4)	24 (100)	144 (79.1)
Focal aware	10 (45.5)	48 (35.3)	7 (29.2)	65 (35.7)
Focal impaired awareness	1 (4.5)	46 (33.8)	12 (50.0)	59 (32.4)
FBTCS	7 (31.8)	76 (55.9)	15 (62.5)	98 (53.8)
Generalized seizures	12 (54.5)	30 (22.1)	1 (4.2)	43 (23.6)
Absence	3 (13.6)	8 (5.9)	0	11 (6.0)
Myoclonic	4 (18.2)	10 (7.4)	0	14 (7.7)
Tonic	0	0	0	0
Atonic	0	0	0	0
Tonic-atonic	2 (9.1)	1 (0.7)	0	3 (1.6)
Clonic	0	1 (0.7)	0	1 (0.5)
Tonic–clonic	10 (45.5)	28 (20.6)	1 (4.2)	39 (21.4)
Epilepsy spasm	0	0	0	0
Other	1 (4.5)	1 (0.7)	0	2 (1.1)
Syndrome, *n* (%)				
*n* ^a^	22	136	24	182
No	12 (54.5)	119 (87.5)	24 (100)	155 (85.2)
Yes				
GTCS only	2 (9.1)	2 (1.5)	0	4 (2.2)
JAE	2 (9.1)	2 (1.5)	0	4 (2.2)
JME	5 (22.7)	8 (5.9)	0	13 (7.1)
Other	1 (4.5)	3 (2.2)	0	4 (2.2)
Etiology, *n* (%)				
*n* ^a^	22	136	24	182
Head injury/cranial trauma	1 (4.5)	8 (5.9)	1 (4.2)	10 (5.5)
CNS infection	0	3 (2.2)	0	3 (1.6)
Stroke	1 (4.5)	7 (5.1)	3 (12.5)	11 (6.0)
Brain structural malformations	3 (13.6)	22 (16.2)	3 (12.5)	28 (15.4)
Vascular brain anomalies	0	3 (2.2)	0	3 (1.6)
Perinatal events	1 (4.5)	4 (2.9)	0	5 (2.7)
Genetic	6 (27.3)	13 (9.6)	0	19 (10.4)
Unknown	10 (45.5)	64 (47.1)	15 (62.5)	89 (48.9)
Other	0	10 (7.4)	2 (8.3)	12 (6.6)
Presence of psychiatric comorbidities, *n* (%)				
*n* ^a^	22	133	24	179
Yes	6 (27.3)	24 (17.6)	3 (12.5)	33 (18.1)
Previous ASMs, *n* (%)				
*n* ^a^	22	136	24	182
At least 1 ASM	8 (36.4)	70 (51.5)	8 (33.3)	86 (47.3)
Most common^c^ previous ASMs, *n* (%)				
*n* ^a^	22	136	24	182
EIASMs				
Carbamazepine	1 (4.5)	20 (14.7)	2 (8.3)	23 (12.6)
Oxcarbazepine	1 (4.5)	2 (1.5)	0	3 (1.6)
Phenobarbital	0	2 (1.5)	0	2 (1.1)
Non-EIASMs				
Lacosamide	0	6 (4.4)	0	6 (3.3)
Lamotrigine	0	8 (5.9)	1 (4.2)	9 (4.9)
Levetiracetam	2 (9.1)	8 (5.9)	6 (25.0)	16 (8.8)
Topiramate	1 (4.5)	2 (1.5)	0	3 (1.6)
Valproate	4 (18.2)	25 (18.4)	0	29 (15.9)
Number of ASMs monotherapies started before baseline, *n* (%)				
n^a^				182
0				1 (0.5)
1				113 (62.1)
≥2				68 (37.4)
Concomitant ASMs, *n* (%)				
*n* ^a^	22	136	24	182
EIASMs	3 (13.6)	25 (18.4)	6 (25.0)	34 (18.7)
Carbamazepine	2 (9.1)	14 (10.3)	2 (8.3)	18 (9.9)
Eslicarbazepine acetate	0	7 (5.1)	1 (4.2)	8 (4.4)
Oxcarbazepine	1 (4.5)	3 (2.2)	3 (12.5)	7 (3.8)
Phenytoin	0	1 (0.7)	0	1 (0.5)
Non-EIASMs	19 (86.4)	111 (81.6)	18 (75.0)	148 (81.3)
Levetiracetam	13 (59.1)	53 (39.0)	12 (50.0)	78 (42.9)
Lamotrigine	3 (13.6)	18 (13.2)	3 (12.5)	24 (13.2)
Valproate	3 (13.6)	17 (12.5)	3 (12.5)	23 (12.6)
Lacosamide	0	14 (10.3)	1 (4.2)	15 (8.2)
Topiramate	0	6 (4.4)	0	6 (3.3)
Clonazepan	0	2 (1.5)	0	2 (1.1)
Zonisamide	0	1 (0.7)	0	1 (0.5)
Brivaracetam	0	1 (0.7)	0	1 (0.5)

^aNumber of PWE for whom datum was available; bInternational League Against Epilepsy 2017 clasisfication. ASM, antiseizure medication; CNS, central nervous system; EIASM, enzyme-inducing antiseizure medication; FBTCS, focal to bilateral tonic–clonic seizures; GTCS, generalized tonic–clonic seizures; JAE, juvenile absence epilepsy; JME, juvenile myoclonic epilepsy; PWE, people with epilepsy; SAS, Safety Analysis Set; SD, standard deviation c>1%.^

### 3.2 ASM treatment

#### 3.2.1 Concomitant ASM treatment

At PER initiation, 62.1% (113/182) of PWE were receiving one ASM monotherapy, 37.4% (68/182) PWE receiving ≥2 ASMs, and one individual was not receiving any concomitant ASMs (Table 2). The majority of concomitant ASMs were non-enzyme-inducing antiseizure medications (EIASMs) (81.3%; 148/182), with the most common being levetiracetam (42.9%), lamotrigine (13.2%), and valproate (12.6%) (Table 2).

Information on the number of concomitant ASMs used at baseline and the end of study was known for 176 PWE: most PWE (86.9%; 153/176) taking one ASM at baseline remained on one ASM at the end of the study; 8.0% (14/176) shifted from one ASM to PER monotherapy; and 4.0% (7/176) shifted from one ASM to two ASMs (plus PER). Only two PWE were receiving two ASMs at baseline: one subject was still down-titrating their previous ASMs while switching to PER, while the other subject was taking two ASMs at baseline (this patient was mistakenly screened as having met all the eligibility criteria, but this error was not picked up until the after the analyses had been performed). Both shifted to one concomitant ASM at the end of the study. At 6 months, 10.1% (17/169) of PWE had a reduction from baseline in concomitant ASM dose, 3.0% (5/169) had an increase, and 1.2% (2/169) had switched to a different ASM. At 12 months, 15.7% (21/134) of PWE had a reduction from baseline in concomitant ASM dose, 7.5% (10/134) an increase, and 3.0% (4/134) had switched to a different ASM.

#### 3.2.2 PER treatment

The mean (SD) daily dose of PER was 4.7 (1.7) mg/day in the overall population, with the daily dose decreasing with age (Supplementary Table S1). Similarly, the mean (SD) modal daily dose of PER was 5.0 (2.0) mg/day overall, with a higher modal dose in younger PWE and reducing with older age (Supplementary Table S1). The most common frequency of PER dose titration was every 2 weeks (in 40.1% of PWE; Supplementary Table S1).

The mean (SD) duration of exposure to PER was 44.9 (17.1) weeks overall, and this was observed to reduce with increasing age (Supplementary Table S1). The duration of exposure was similar in PWE with focal-onset seizures and in those with GTCS (Supplementary Table S1). The duration of exposure for PER by baseline ASM is summarized in Supplementary Table S1.

### 3.3 Effectiveness assessments

#### 3.3.1 Primary endpoint—retention rate at 12 months

**Figure 3:**
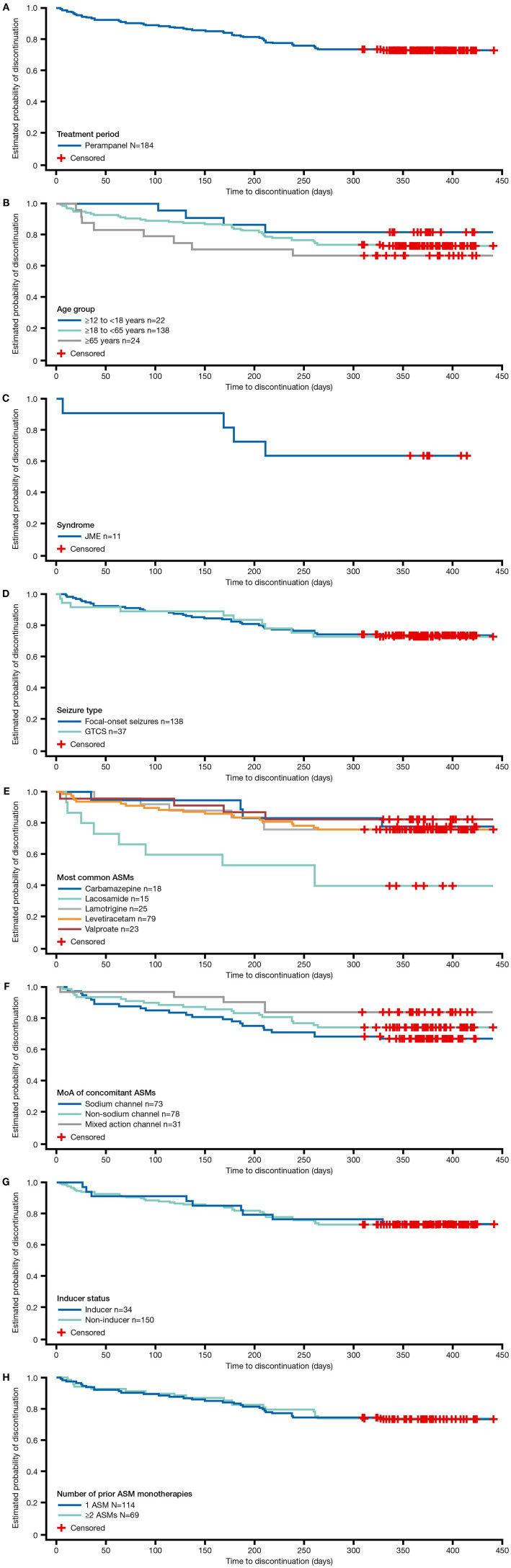
Kaplan–Meier plots of retention over 12 months: **(A)** overall population, **(B)** subgroups of PWE aged 12 to <18, ≥18 to <65, and ≥ 65 years, **(C)** by syndrome, **(D)** by seizure type, **(E)** by most common ASM monotherapy at baseline, **(F)** by MoA of baseline ASMs monotherapy, **(G)** by inducer status, and **(H)** by number of previous monotherapies. ASM, antiseizure medication; GTCS, generalized tonic–clonic seizures; JME, juvenile myoclonic epilepsy; MoA, mechanism of action; PER, perampanel.

The retention rate at 12 months was 74.2% (135/182; 95% CI: 67.8, 80.5%) overall and decreased with age from 81.8% (18/22; 95% CI: 65.7, 97.9%) in the 12 to <18 years age group, to 74.3% (101/136; 95% CI: 66.9, 81.6) in the 18 to <65 years age group, and to 66.7% (16/24; 95% CI: 47.8, 85.5%) in the ≥65 years age group (Figure 3; Tables 3, 4). Retention rates were similar between PWE with focal-onset seizures (74.5% [102/137; 95% CI: 67.1, 81.8%]) and GTCS (75.0% [27/36; 95% CI: 60.9, 89.1%]). In PWE with JME, 63.6% (7/11; 95% CI: 35.2, 92.1%) were still on PER at 12 months (Table 4).

**Table 3:** Summary of key efficacy endpoints in the overall population.

	Efficacy outcome in the overall population	
	6 months	12 months
Retention rate, % (95% CI); n/N	83.0% (77.5, 88.4%); 151/182	74.2 (67.8, 80.5); 135/182
Change in seizure frequency per 28 days from baseline (all seizures) Median reduction (95% CI)	−73.3 (−92.9, −51.3)	−78.6 (−100.0, −66.3)
Pragmatic seizure freedom rate, % (95% CI); n/N	37.9 (30.7, 45.1); 66/174	36.2 (29.1, 43.3); 63/174
Completers seizure freedom rate, % (95% CI); n/N	44.0 (36.1, 51.9); 66/150	46.7 (38.3, 55.1); 63/150
Responder rate, % (95% CI); n/N	60.3 (54.8, 69.4); 105/174	64.4 (59.1, 73.4); 112/174
Worsening rate, % (95% CI); n/N	9.8 (5.4, 14.2); 17/174	8.6 (4.5, 12.8); 15/174

CI, confidence interval.

**Table 4:** Summary of PER key effectiveness assessments in different subgroups: retention rates at 12 months (primary endpoint), pragmatic seizure freedom rate at 6 and 12 months, and completer seizure freedom at 6 and 12 months in the overall population and different subgroups.

Population	Retention at 12 months (primary endpoint)	Pragmatic seizure freedom rate		Completers seizure freedom rate	
		6 months	12 months	6 months	12 months
Age group, % (95% CI); n/N					
12 to <18 years	81.8 (65.7, 97.9); 18/22	52.4 (31.0, 73.7); 11/21	38.1 (17.3, 58.9); 8/21	61.1 (38.6; 83.6); 11/18	44.4 (21.5, 67.4); 8/18
18 to <65 years	74.3 (66.9, 81.6); 101/136	36.4 (28.2, 44.6); 48/132	34.8 (26.7, 43.0); 46/132	41.7 (32.7; 50.8); 48/115	45.5 (35.8, 55.3); 46/101
≥65 years	66.7 (47.8, 85.5); 16/24	33.3 (13.2, 53.5); 7/21	42.9 (21.7, 64.0); 9/21	41.2 (17.8; 64.6); 7/17	56.3 (31.9, 80.6); 9/16
Seizure type, % (95% CI); n/N					
Focal-onset	74.5 (67.1, 81.8); 102/137	34.8 (26.7, 43.0); 46/132	34.1 (26.0, 42.2); 45/132	40.7 (31.6; 49.8); 46/113	44.1 (34.5, 53.8); 45/102
Focal aware motor onset		35.3; 6/17	23.5; 4/17	42.9; 6/14	28.6; 4/14
Focal aware non-motor onset		25.0; 5/20	15.0; 3/20	29.4; 5/17	17.6; 3/17
Focal impaired awareness		15.9; 7/44	11.4; 5.44	18.4; 7/38	13.2; 5/38
FBTCS		37.5; 15/40	30.0; 12/40	39.5; 15/38	31.6; 12/38
Generalized-onset					
Tonic–clonic in IGE^a^	75.0 (60.9, 89.1); 27/36	54.5 (37.6, 71.5); 18/33	45.5 (28.5, 62.4); 15/33	60.0 (42.5, 77.5); 18/30	55.6 (36.8, 74.3); 15/27
Absence		16.7; 2/12	33.3; 4/12	20.0; 2/10	40.0; 4/10
Atypical		0	0	0	0
Myoclonic		54.5 (6/11)	54.5; 6/11	54.5; 6/11	54.5; 6/11
Tonic		33.3 (1/1)	66.7; 2/3	33.3; 1/3	66.7; 2/3
Atonic		0	0	0	0
Tonic-Atonic		0	0	0	0
Clonic		100; 1/1	100.0; 1/1	100; 1/1	100; 1/1
Tonic–clonic		39.1 (9/23)	39.1; 9/23	42.9; 9/21	42.9; 9/21
Epilepsy spasm		0	0	0	0
Other		0	0	0	0
Syndrome, % (95% CI); n/N					
JME	63.6 (35.2, 92.1); 7/11	55.6 (23.1, 88.0); 5/9	44.4 (12.0, 76.9); 4/9	71.4 (38.0, 104.9); 5/7	57.1 (10.5, 93.8); 4/7
Most common ASM at baseline, % (95% CI); n/N					
CBZ	77.8 (58.6, 97.0); 14/18	27.8 (7.1, 48.5); 5/18	27.8 (7.1, 48.5); 5/18	29.4 (7.8, 51.1); 5/17	35.7 (10.6, 60.8); 5/14
LCM	40.0 (15.2; 64.8); 6/15	21.4 (0; 42.9); 3/14	14.3 (0; 32.6); 2/14	37.5 (4.0, 71.0); 3/8	33.3 (0, 71.1); 2/6
LTG	79.2 (62.9, 95.4); 19/24	52.2 (31.8, 72.6); 9/23	39.1 (19.2, 59.1); 9/23	60.0 (38.5, 81.5); 12/20	47.4 (24.9, 69.8); 9/19
LEV	76.9 (67.6, 86.3); 60/78	39.2 (28.1, 50.3); 29/74	41.9 (30.7, 53.1); 31/74	43.9 (32.0, 75.1); 10/16	51.7 (39.0, 64.3); 31/60
VPA	82.6 (67.1, 98.1); 19/23	45.5 (24.6, 66.3); 10/22	40.9 (20.4, 61.5); 9/22	52.2 (30.2, 75.1); 10/19	47.4 (24.9, 69.8); 9/19
MoA of baseline ASM^b^, % (95% CI); n/N					
Sodium channel blocker	68.1 (57.3, 78.8); 49/72	33.3 (22.2, 44.5); 23/69	29.0 (18.3, 39.7); 20/69	41.1 (28.2, 54.0); 23/56	40.8 (27.1, 54.6); 20/49
Non-sodium channel blocker	75.3 (65.7, 85.0); 58/77	38.4 (27.2. 49.5); 28/73	39.7 (28.5, 51.0); 29/73	43.1 (31.0, 55.1); 28/65	50.0 (37.1, 62.9); 28/58
Mixed-action	83.9 (70.9, 96.8); 26/31	43.3 (25.6, 61.1); 13/30	40.0 (22.5, 57.5); 12/30	48.1 (29.3, 67.0); 13/27	46.2 (27.0, 65.3); 12/26
Inducer status of concomitant ASM, % (95% CI); n/N					
EIASMs	73.5 (58.7, 88.4); 25/34	27.3 (12.1, 42.5); 9/33	30.3 (14.6, 46.0); 10/33	31.0 (14.2, 47.9); 9/29	40.0 (20.8, 59.2); 10/25
Non-EIASMs	74.3 (67.3, 81.4); 110/148	40.4 (32.3, 48.5); 57/141	37.6 (29.6, 45.6); 53/141	47.1 (38.2, 56.0); 57/121	48.2 (38.8, 57.5); 53/110
Number of previous ASMs, % (95% CI); n/N					
1	74.3 (66.3, 82.4); 84/113	41.5 (32.1, 50.9); 44/106	44.3 (34.9, 53.8); 47/106	47.3 (37.2, 57.5); 44/93	56.0 (45.3, 66.6); 47/84
≥2	75.0 (64.7; 85.3); 51/68	32.8 (21.6; 44.1); 22/67	23.9 (13.7; 34.1); 16/67	38.6 (26.0, 51.2); 22/57	31.4 (18.6, 44.1); 16/51
Seizure etiology^c^, % (95% CI); n/N					
Genetic	78.9 (60.6, 97.3); 15/19	50.0 (26.9, 73.1); 9/18	33.3 (11.6, 55.1); 6/18	56.3 (31.9, 80.6); 9/16	40.0 (15.2, 64.8); 6/15
Structural	77.8 (65.6, 89.9); 35/45	37.2 (22.8, 51.7); 16/43	44.2 (29.3, 59.0); 19/43	42.1 (26.4, 57.8); 16/38	54.3 (37.8, 70.8); 19/35
Other etiologies	66.7 (40.0, 93.3); 8/12	18.2 (−4.6, 41.0); 2/11	45.5 (16.0, 74.9); 5/11	25.0 (−0.5, 55.0); 2/8	62.5 (29.0, 96.0); 5/8

^aIncluded PWE with a history of tonic–clonic seizures associated with IGE as per ILAE definition and no history of focal seizures; bSodium channel blocker: carbamazepine, lamotrigine, oxcarbazepine, phenytoin, lacosamide, rufinamide, eslicarbazepine, and mephenytoin; non-sodium channel blocker: levetiracetam, pregabalin, gabapentin, tiagabine, ethosuximide, acetazolamide, retigabine, and brivaracetam; mixed action: valproate, topiramate, clobazam, clonazepam, zonisamide, phenobarbital, primidone, and felbamate. cGenetic etiology: defined genetic cause; structural etiology: CNS infection, stroke, structural brain anomalies or malformations or vascular brain anomalies; other etiologies: all other etiologies.^

ASM, antiseizure medication; CBZ, carbamazepine; CI, confidence interval; EIASMs, enzyme-inducing antiseizure medications; FBTCS, focal to bilateral tonic–clonic seizures; IGE, idiopathic generalized epilepsy; ILAE, International League Against Epilepsy; JME, Juvenile myoclonic epilepsy; LCM, lacosamide; LEV, levetiracetam; LTG, lamotrigine; MoA, mechanism of action; VPA, valproate.

The retention rate ranged from 40% (6/15; 95% CI: 15.2, 64.8%) in PWE receiving concomitant lacosamide and 82.6% (19/23; 95% CI: 67.1, 98.1%) in those receiving concomitant valproate. Concomitant administration with a mixed-action ASM was associated with higher retention rates (83.9%) compared with non-sodium channel blocker and sodium channel blocker ASMs (75.3 and 68.1%, respectively), but no difference was observed for PWE receiving concomitant EIASMs versus non-EIASMs (73.5% vs. 74.3%). PWE, with all other etiologies, had a lower retention rate than PWE with genetic and structural etiology (66.7, 78.9, and 77.8%, respectively). Retention rates were similar between PWE who received one versus ≥2 prior ASMs (74.3% vs. 75.0%) (Table 4).

The multivariate analysis indicated that age at diagnosis was found to influence retention, with a younger age at diagnosis resulting in better retention (*p* = 0.0096) (Supplementary Table S2).

#### 3.3.2 Secondary endpoints—retention rates at 6 months

The retention rate at 6 months was 83.0% (151/182; 95% CI: 77.5, 88.4%) for the overall population, 86.4% (19/22; 95% CI: 72.0, 100.7%) in PWE aged 12 to <18 years, 84.6% (115/136; 95% CI: 78.5, 90.6%) in those aged 18 to <65 years, and 70.8% (17/24; 95% CI: 52.6, 89.0%) in those aged ≥65 years (Table 3). The retention rate at 6 months was 82.5% (113/137; 95% CI: 76.1, 88.8%) in PWE with focal-onset seizures and 86.1 (31/36; 95% CI: 74.8, 97.4%) in those with GTCS.

#### 3.3.3 Secondary endpoints—change in seizure frequency at 6 and 12 months

The overall median reduction in monthly seizure frequency per 28 days from baseline to 6 months was −73.3% (95% CI: −92.9, −51.3%) in the overall population, −100.0% (95% CI: −100.0, −66.67%) in the 12 to <18 age group, −66.7% (95% CI: −83.5 to −50.0%) in the 18 to <65 years age group, and − 70.8% (95% CI: −100.0 to −0.0%) in the ≥65 years age group (Table 3; Figure S1A). Values for the 12-month timepoint in the respective groups were − 78.6% (95% CI: −100.0, −66.3%), −92.3% (95% CI: −100.0 to −54.4%), −75.0% (95% CI: −100.0, −64.2%), and − 87.5% (95% CI: −100.0 to −25.0%) (Table 3; Figure S1A). Overall, there was a reduction in monthly seizure frequency across all seizure types at both 6 and 12 months (Figures S1B, C).

#### 3.3.4 Secondary endpoints: pragmatic seizure freedom rate at 6 and 12 months

In the overall population, the pragmatic seizure freedom rate was 37.9% (66/174; 95% CI: 30.7, 45.1%) at 6 months and 36.2% (63/174; 95% CI: 29.1, 43.3%) at 12 months (Figure S2; Tables 3, 4). In the 12 to <18, the 18 to <65, and the ≥65 years age groups, the pragmatic seizure freedom rates were 52.4, 36.4, and 33.3%, respectively, at 6 months and 38.1, 34.8, and 42.9%, respectively, at 12 months (Table 4). At both time points, the pragmatic seizure freedom rate was higher in the subgroup with generalized seizures than in the subgroup with focal-onset seizures (Table 4). It ranged between 16 and 38% at 6 months and between 11 and 30% at 12 months for all focal-onset seizure types (with the highest rate being achieved for FBTCS) and between 17 and 100% at 6 months and between 33 and 100% at 12 months for all generalized-onset seizure types (Table 4). The seizure freedom rate in PWE with JME was 55.6% (5/9; 95% CI: 23.1, 88.0%) at 6 months and 44.4% (4/9; 95% CI: 12.0, 76.9%) at 12 months.

Seizure freedom rates in the subgroups of PWE receiving different ASM monotherapy ranged between 21.4 and 52.2% at 6 months and between 14.3 and 41.9% at 12 months (Table 4). The proportion of PWE who achieved seizure freedom was higher when PER was administered with a non-sodium channel blocker ASM (38.4% at 6 months and 39.7% at 12 months) and a mixed-action ASM (43.3% at 6 months and 40.0% at 12 months) than with a sodium channel blocker ASM (33.3% at 6 months and 29.0% at 12 months), and with a non-EIASM (6 months: 40.4%; 12 months: 37.6%) than with an EIASM (6 months: 27.3%; 12 months: 30.3%) (Table 4). At 6 months, the pragmatic seizure freedom rate was lowest in the subgroup with other etiologies and highest in the subgroup with genetic etiology (18.2% vs. 50.0%), but the opposite was observed at 12 months (45.5% vs. 33.3%). At both 6 and 12 months, the seizure freedom rate was higher in those PWE who had received only one previous ASM monotherapy (41.5% at 6 months and 44.3% at 12 months) than in those who had received ≥2 previous monotherapies (32.8% at 6 months and 23.9% at 12 months) (Table 4).

According to the sensitivity analysis, the pragmatic seizure freedom rate was 36.3% (66/182; 95% CI: 29.3, 43.2%) at 6 months and 34.6% (63/182; 95% CI: 27.7, 41.5%) at 12 months.

The number of previous ASMs influenced the likelihood of achieving seizure freedom, with PWE who had taken one previous ASM more likely to be seizure-free than those who had taken ≥2 previous ASMs both in the univariate (odds ratio [OR]: 0.39; 95% CI: 0.20, 0.78; *p* = 0.0072) and the multivariate (OR: 0.41; 95% CI: 0.185, 0.884; *p* = 0.0232) analyses (Supplementary Table S2).

#### 3.3.5 Secondary endpoints: completers seizure freedom rate at 6 and 12 months

In the overall population, the completers seizure freedom rates at 6 and 12 months were 44.0% (66/150; 95% CI: 36.1, 51.9%) and 46.7% (63/150; 95% CI: 38.3, 55.1%), respectively (Figure S2; Tables 3, 4). At 6 months, the completers seizure freedom rates were 61.1, 41.7, and 41.2% in the 12 to <18, 18 to <65, and ≥ 65 years age groups, respectively, and the corresponding values at 12 months were 44.4, 45.5, and 56.3%, respectively (Table 4). The completers seizure freedom rate was higher in the subgroup with generalized-onset seizures than in the subgroup with focal-onset seizures at both 6 months (60.0% vs. 40.7%) and 12 months (55.6% vs. 44.1%), ranging between 18 and 43% at 6 months and between 13 and 32% at 12 months for all focal-onset seizure types and between 20 and 100% at 6 months and between 40 and 100% at 12 months for all generalized-onset seizure types (Table 4).

The completers seizure freedom rate in PWE with JME was 71.4% (5/7; 95% CI: 38.0, 100%) at 6 months and 57.1% (4/7; 95% CI: 20.5, 93.8%) at 12 months. Results for completers seizure freedom rates were similar to those reported for pragmatic seizure freedom rates. Higher seizure freedom rates at 6 and 12 months were achieved when PER was administered with a non-sodium channel blocker ASM and a mixed-action ASM than with a sodium-channel blocker; with a non-EIASM than with an EIASMs; and in those PWE who had received only one previous ASM monotherapy than in those who received ≥2 ASM monotherapy (Table 4). Results for the subgroups with different etiologies (genetic, structural, and other etiologies) were similar to the ones observed for the pragmatic seizure freedom rates (Table 4).

According to the sensitivity analysis, completers seizure freedom rate was 43.7% (66/151; 95% CI: 35.8, 51.6%) at 6 months and 46.7% (63/151; 95% CI: 38.3, 55.1%) at 12 months.

The number of previous ASMs influenced seizure freedom with PWE taking one previous ASM more likely to be seizure-free than those taking ≥2 previous ASMs both in the univariate (OR: 0.360; 95% CI: 0.173, 0.748; *p* = 0.0062) and the multivariate (OR: 0.367; 95% CI: 0.150, 0.899; *p* = 0.0284) analyses (Supplementary Table S2). However, in the univariate analysis, age at epilepsy diagnosis was found to influence seizure occurrence, with a younger age at diagnosis being associated with a higher likelihood of achieving seizure freedom than an older age at diagnosis (OR: 1.0; 95% CI: 1.000, 1.039; *p* = 0.0277).

#### 3.3.6 Secondary endpoint: responder rate and seizure worsening rate

In the overall population, the responder rate was 60.3% (105/174; 95% CI: 54.8, 69.4%) at 6 months and 64.4% (112/174; 95% CI: 59.1, 73.4%) at 12 months (Table 3). At 6 and 12 months, the responder rate was 90.5% (19/21; 95% CI: 77.9, 103%) and 76.2% (16/21; 95% CI: 58.0, 94.4%), respectively, in the 12 to <18 years age group, 56.1% (74/132; 95% CI: 49.3, 66.4%) and 62.9% (83/132; 95% CI: 56.6, 73.1%), respectively, in the 18 to <65 years age group, and 57.1% (12/21; 95% CI: 38.5, 81.5%) and 61.9% (13/21; 95% CI: 44.1, 85.9%), respectively, in the ≥65 years age group. Responder rates by detailed seizure types (Figure S2; Supplementary Table S3) ranged from 46 to 70% for all focal-onset seizure types and from 58 to 100% for all generalized-onset seizures types at 6 months, and from 55 to 78% for focal-onset seizure types and from 65 to 100% for generalized-onset seizure types at 12 months.

The seizure worsening rate was 9.8% (17/174; 95% CI: 5.4, 14.2%) at 6 months and 8.6% (15/174; 95% CI: 4.5, 12.8%) at 12 months in the overall population (Table 3). Corresponding values in the different age subgroups were 0 and 9.5% (2/21; 95% CI: −3.0, 22.1%) in the 12 to <18 years subgroup, 9.8% (13/132; 95% CI: 4.8, 14.9%) and 9.1% (12/132; 95% CI: 4.2, 14.0%) in the 18 to <65 years subgroup, and 19.0% (4/21; 95% CI: 2.3, 35.8%) and 4.8% (1/21; 95% CI: −4.3, 13.9%) in the ≥65 years subgroup. Seizure worsening rates by detailed seizure types are reported in Supplementary Table S3. At 6 months, seizure worsening rates ranged between 8 and 18% for focal-onset seizures and between 8 and 17% for generalized seizures; at 12 months, they ranged between 5 and 18% for focal-onset seizures and only 4 PWE with generalized-onset seizures (17.4%) had seizure worsening.

During the 12-month study, 4.9% (9/182) of PWE experienced status epilepticus/convulsions, with the proportion being higher in the youngest and oldest age groups (9.1% [2/22], 3.7% [5/136], and 8.3% [2/24] in the 12 to <18, 18 to <65, and ≥ 65 age groups, respectively).

#### 3.3.7 Exploratory endpoints: QOLIE-10, ESS, EpiTrack, and EpiTrack Junior

Only five adults (≥18 years) had QOLIE-10 assessments at baseline and 6 months: the mean (SD) change in QOLIE-10 total score at 6 months relative to baseline was −0.02 (0.531; 95% CI: −0.68, 0.64) in both the observed case and LOCF analyses. At 12 months, the mean (SD) change of QOLIE-10 total score at 12 months relative to baseline was −0.82 (0.942; 95% CI: −1.99, 0.35; *n* = 5) in the observed case analysis and − 0.54 (0.894; 95% CI: −1.29, 0.21; *n* = 8) in the LOCF analysis.

In the adult population, ESS scores were reported for 16 PWE at 6 months and the mean (SD; 95% CI) change from baseline was −0.6 (3.72; −2.5, 1.4) in the observed case analysis and the LOCF analysis. Changes from baseline in ESS score at 12 months were − 0.1 (2.87; −1.7, 1.4; *n* = 16) in the observed case analysis and 0.3 (3.48; −1.3, 1.9; *n* = 21) in the LOCF analysis.

In the adult subgroup (*n* = 7), the mean (SD; 95% CI) change in EpiTrack age-corrected total score from baseline to 6 months was −1.6 (5.50; −6.7, 3.5) in both the observed case analysis and LOCF analysis. The mean (SD; 95% CI) change in EpiTrack age-corrected total score from baseline to 12 months was 0.8 (2.87; 95% CI: −3.8 to 5.3; *n* = 4) in the observed case analysis and − 0.6 (4.72; 95% CI: −4.9 to 3.8; *n* = 7) in the LOCF analysis. In the adolescent subgroup (≥12 to <18 years, *n* = 4), the mean (SD; 95% CI) change in EpiTrack Junior age-corrected total score was −0.8 (3.86; 95% CI: −6.9 to 5.4) both in the observed case and LOCF analyses at 6 months relative to baseline, and − 1.3 (2.08; 95% CI: −6.5, 3.8; *n* = 3) in the observed case analysis and − 1.8 (1.89; 95% CI: −4.8, 1.3; *n* = 4) in the LOCF analysis at 12 months relative to baseline.

### 3.4 Safety and tolerability

Overall, 52.7% (96/182) of PWE experienced TEAEs, with the incidence decreasing with age being 63.6% (14/22) in the 12 to <18 years group, 51.5% (70/136) in the 18 to <65 years age group, and 50.0% (12/24) in the ≥65 years group (Table 5). Treatment-related TEAEs were experienced by 33.0% (60/182) of the total population, with the youngest age group experiencing the highest proportion of treatment-related TEAEs (63.6% [14/22]) compared to the other age groups. Most TEAEs were of mild or moderate intensity and 10 (5.5%) PWE experienced severe TEAEs. The proportion of PWE who underwent weekly PER titration was higher in the subgroup of patients who reported TEAEs than in the subgroup who did not report TEAEs (29.2% vs. 18.6%). Conversely, the proportions of patients who underwent a 2-week (45.3% vs. 35.4%) and 4-week (12.6% vs. 6.2%) titration pattern were higher in the subgroup of PWE who did not report TEAEs than in the subgroup who did report TEAEs. The most common TEAEs overall were dizziness (10.4%), irritability (8.8%), and somnolence (8.2%) (Table 5). Serious TEAEs were reported for 6.0% (11/182) of the total population. Overall, 36/182 (19.8%) PWE experienced at least one psychiatric TEAE: of these, 23 had no history of psychiatric events and 13 did. The most common psychiatric disorder TEAEs were irritability (8.8%), insomnia (3.3%), aggression (2.7%), anxiety (1.6%), and behavioral disorder (1.6%).

**Table 5:** Summary of safety and tolerability of PER (SAS).

	Overall population (*N* = 182)	12 to < 18 years age group (*n* = 22)	18 to < 65 years age group (*n* = 136)	≥65 years age group (*n* = 24)
Any TEAEs, *n* (%)	96 (52.7)	14 (63.6)	70 (51.5)	12 (50.0)
Treatment-related TEAEs, *n* (%)	60 (33.0)	14 (63.6)	39 (28.7)	7 (29.2)
Most common^a^ TEAEs, *n* (%)				
Dizziness	19 (10.4)	6 (27.3)	10 (7.4)	3 (12.5)
Irritability	16 (8.8)	5 (22.7)	10 (7.4)	1 (4.2)
Somnolence	15 (8.2)	6 (27.3)	8 (5.9)	1 (4.2)
Nasopharyngitis	13 (7.1)	0	12 (8.8)	1 (4.2)
Vertigo	11 (6.0)	1 (4.5)	9 (6.6)	1 (4.2)
Headache	9 (4.9)	0	8 (5.9)	1 (4.2)
Seizures worsening	9 (4.9)	2 (9.1)	5 (3.7)	2 (8.3)
Severe TEAEs, *n* (%)	10 (5.5)	1 (4.5)	6 (4.4)	3 (12.5)
Serious TEAEs, *n* (%)	11 (6.0)	1 (4.5)	6 (4.4)	4 (16.7)
Death	4 (2.2)	0	2 (1.5)	2 (8.3)
Others	7 (3.8)	1 (4.5)	4 (2.9)	2 (8.3)
Life-threatening	0	0	0	0
Requires inpatient hospitalization or prolongation of existing hospitalization	7 (3.8)	1 (4.5)	4 (2.9)	2 (8.3)
Persistent or significant disability or incapacity	1 (0.5)	1 (4.5)	0	0
Congenital anomaly/birth defect	0	0	0	0
Important medical events	0	0	0	0
TEAEs leading to PER dose adjustment, *n* (%)	36 (19.8)	5 (22.7)	25 (18.4)	6 (25.0)
TEAEs leading to PER withdrawal	22 (12.1)	2 (9.1)	14 (10.3)	6 (25.0)
TEAEs leading to PER dose increase	4 (2.2)	2 (9.1)	2 (1.5)	0
TEAEs leading to PER dose reduction	14 (7.7)	2 (9.1)	12 (8.8)	0
TEAEs leading to PER dose interruption	3 (1.6)	0	3 (2.2)	0

By concomitant ASM monotherapy					
	CBZ (*n* = 18)	LCM (*n* = 15)	LTG (*n* = 24)	LEV (*n* = 78)	VPA (*n* = 23)
Any TEAEs	8 (44.4)	6 (40.0)	13 (54.2)	43 (55.1)	12 (55.2)
Treatment-related TEAEs, *n* (%)	5 (27.8)	3 (20.0)	7 (29.2)	28 (35.9)	8 (34.8)
Severe TEAEs, *n* (%)	1 (5.6)	2 (13.3)	0	5 (6.4)	1 (4.3)
Serious TEAEs, *n* (%)	1 (5.6)	2 (13.3)	0	6 (7.7)	1 (4.3)
Death	0	2 (13.3)	0	2 (2.6)	0
Others	1 (5.6)	0	0	4 (5.1)	1 (4.3)
Life threatening	0	0	0	0	0
Requires inpatient hospitalization or prolongation of existing hospitalization	1 (5.6)	0	0	4 (5.1)	1 (4.3)
Persistent or significant disability or incapacity	0	0	0	1 (1.3)	0
Congenital anomaly/birth defect^b^	0	0	0	0	0
TEAEs leading to study PER dose adjustment, *n* (%)	2 (11.1)	4 (26.7)	5 (20.8)	14 (17.9)	4 (17.4)
TEAEs leading to PER dose withdrawal	1 (5.6)	3 (20.0)	3 (12.5)	10 (12.8)	1 (4.3)
TEAEs leading to PER dose reduction	1 (5.6)	1 (6.7)	2 (8.3)	4 (5.1)	3 (13.0)
TEAEs leading to PER dose interruption	1 (5.6)	0	0	1 (1.3)	0

By PER maximum dose			
	≤4 mg (*n* = 77)	4 to < 7 mg (*n* = 66)	8–12 mg (*n* = 39)
Any TEAEs	37 (48.1)	37 (56.1)	22 (56.4)
Treatment-related TEAEs, *n* (%)	26 (33.8)	19 (28.8)	15 (38.5)
Severe TEAEs, *n* (%)	3 (3.9)	3 (4.5)	4 (10.3)
Serious TEAEs, *n* (%)	3 (3.9)	4 (6.1)	4 (10.3)
Death	2 (2.6)	2 (3.0)	0
Others	1 (1.3)	2 (3.0)	4 (10.3)
Life threatening	0	0	0
Requires inpatient hospitalization or prolongation of existing hospitalization	1 (1.3)	2 (3.0)	4 (10.3)
Persistent or significant disability or incapacity	0	0	1 (2.6)
Congenital anomaly/birth defect	0	0	0
Important medical events	0	0	0
TEAEs leading to PER dose adjustment, *n* (%)	22 (28.6)	10 (15.2)	4 (10.3)
TEAEs leading to PER dose withdrawal	17 (22.1)	5 (7.6)	0
TEAEs leading to PER dose reduction	5 (6.5)	5 (7.6)	4 (10.3)
TEAEs leading to PER dose interruption	2 (2.6)	1 (1.5)	0

^aReported by >5% in any age group; bPregnancies were recorded for four individuals: no safety data were reported for three and one individual continued PER during pregnancy and gave birth to a healthy girl via cesarean section without adverse events.^

ASM, antiseizure medication; CBZ, carbamazepine; LCM, lacosamide; LEV, levetiracetam; LTG, levetiracetam; PER, perampanel; SAS, Safety Analysis Set; TEAE, treatment-emergent adverse event; VPA, valproate.

No TEAEs in relation to dyslipidemia, hypercholesterolemia, or hyperlipidemia were reported. Four deaths occurred, none of which were considered related to PER: two deaths in each of the older age groups and none in the youngest age group (Table 5). No safety concerns relating to either suicidality or falls were reported. No changes of clinical importance in weight or body mass index over time were observed.

TEAEs led to dose adjustment and PER discontinuation in 19.8% (36/182) and 12.1% (22/182), respectively, of PWE in the total population, with the highest proportions of PER adjustments and discontinuations (25.0 and 25.0%, respectively) occurring in the oldest age group (Table 5).

The proportion of TEAEs varied by concomitant ASM from 55.1% (43/78) in PWE treated with concomitant LEV to 40.0% (6/15) in those receiving concomitant LCM (Table 5). TEAEs were reported in 51.0% (50/98) of PWE receiving a modal dose of PER ≤4 mg/day, in 49.2% (29/59) of those receiving 4 to <8 mg, and in 68.0% (17/25) of those receiving 8–12 mg/day (Table 5). In the group of PER ≤4 mg/day, the majority of PWE (86.0%) were receiving a concomitant non-EIASM (most commonly levetiracetam [36.0%], lamotrigine [20.0%], lacosamide [12.0%], and valproate [12.0%]).

Alertness- and cognition-related TEAEs were experienced by 18.7% (34/182) of PWE overall, most commonly somnolence (8.2%), aggression (2.7%), and behavior disorder (1.6%). A higher proportion of alertness- and cognition-related TEAEs were experienced by the younger age group (36.4% [8/22]) than in the older age groups (15.4% [21/136] and 20.8% [5/24], respectively).

## 4 Discussion

In this prospective, observational study, PER used as early add-on therapy demonstrated high retention rates, with approximately three-quarters of PWE overall still receiving PER at the end of the 12-month study period, and high retention rates being reported for both focal-onset seizures and GTCS. Among baseline characteristics, only age at diagnosis was found to influence retention rates, with a younger age at diagnosis resulting in better retention at 12 months.

Analysis of seizure-related endpoints indicated that PER effectively reduced the occurrence of seizures: over 12 months of PER treatment, approximately 60% of PWE responded to PER treatment and over one-third achieved seizure freedom. These results are in line with the findings observed with the second- or third-line treatment regimen with different ASMs in a 30-year longitudinal study of treatment outcomes in 1795 people with newly diagnosed epilepsy (3). The difference in seizure freedom rates noted with different types of concomitant ASMs might be related to the different mechanisms of action of concomitant ASMs, as different ASMs may have synergistic effects (23), suggesting that PER’s unique mechanism of action as first-in-class non-competitive selective AMPA receptor antagonist provides a valuable option to achieve rational polytherapy (24, 25).

PER was efficacious for both focal-onset and generalized seizures, supporting evidence from the extensive clinical trial program (8–11). However, seizure freedom rates and responder rates were higher in PWE with generalized seizures, including absence seizures, myoclonic seizures, tonic seizures, clonic seizures, and tonic–clonic seizures, than in those with focal-onset seizures, supporting the use of PER as a broad-spectrum ASM (26, 27). Logistic regression analyses indicated that the number of previous ASMs and age of epilepsy onset might influence seizure freedom, with PWE taking one previous ASM more likely to be seizure-free than those taking two or more previous ASMs, and with a younger age at diagnosis resulting in a higher probability of achieving seizure freedom. This is likely related to the fact that patients treated with ≥2 previous ASMs are likely to be more refractory to treatment and/or later in their disease course than those treated with one previous ASM.

The PERMIT Extension study was a large, pooled analysis of PER clinical practice studies, which evaluated PER in the total population and by age category (<12, ≥12 to <18, ≥18 to <65, and ≥ 65 years at baseline). PER was shown to be effective and generally well-tolerated when used to treat people with focal or generalized epilepsy, regardless of age category (28), but its effectiveness was greatest in PWE aged ≥65 years. The incidence of AEs was also higher in PWE aged ≥65 years than the other age categories due to factors such as age-related changes in drug pharmacokinetics and pharmacodynamics, higher prevalence of comorbidities, and greater use of polypharmacy. Similar findings were also reported in the Follow-up of 1 Year Data of paTients on perAmpanel (FYDATA) study, in which patients aged ≥65 years, those with epilepsy due to a vascular etiology, and those who had received fewer prior ASMs showed a better clinical response to PER (29). In the current study, PER was efficacious across all age categories, but older PWE reported lower retention rates and higher discontinuations due to TEAEs, which is in line with the results reported in the PERMIT Extension study (28).

Safety and tolerability findings were consistent with the known safety profile of PER (5, 6), with dizziness, irritability, somnolence, and vertigo being the most commonly reported TEAEs. No new safety concerns were identified with PER as a first add-on treatment, and no TEAEs related to either suicidality or falls were reported.

Previous observational studies suggested that PER is an efficacious early add-on therapy, and Study 512 provides further evidence of its effectiveness in this setting. In a multicenter, retrospective, 1-year observational study of 149 individuals with focal epilepsy or IGE aged ≥12 years who initiated PER as the first add-on therapy, 45.6% of PWE were seizure-free and 84.6% were responders (defined as ≥50% reduction in seizure frequency at 12 months since at least during the last 6 months) (12). In the current study, pragmatic seizure freedom at 12 months was 36.2% and responder rate was 64.4%. In the interim analysis of the multicenter, prospective, observational, non-interventional PERPRISE study (Study 509), PER was administered as the only add-on therapy to ASM monotherapy or as a substitute for one ASM in dual therapy in adults with FBTCS or GTCS (30). The analysis included 100 PWE, and at 6 months, the retention rate was 78.0%, the seizure freedom rate 58.8%, and the 50% responder rate 82.6%. In the current study, at 6 months, the retention rate was 83.0%; the pragmatic seizure freedom was 37.9% for all seizures, 37.5% for FBTCS, and 54.5% for GTCS; and the responder rate was 60.3% for all seizures. TEAEs were reported by 48.0% of individuals included in the PERPRISE study versus 52.7% in the current study. The AMPA study evaluated the effectiveness and safety of adjunctive PER in patients aged >12 years with focal-onset seizures (with or without FBTCS) in the clinical practice setting in Italy (31). Retention rates were 72.6% at 6 months and 57.3% at 12 months, compared to 83.0 and 74.2%, respectively, in the current study. At 6 and 12 months, seizure freedom rates for all seizures were 18.0 and 18.8%, respectively; corresponding values for FBTCS were up to 61.3 and 56.3%, respectively. In the current study, pragmatic seizure freedom rates were 34.8% at 6 months and 34.1% at 12 months for all focal seizures and 37.5% at 6 months and 30.0% at 12 months for FBTCS. TEAEs were reported by 56.4% of PWE (vs. 52.7% in the current study) (31).

A few studies have also compared the effects of PER when used as early versus late add-on therapy, suggesting that PER might be more efficacious and better tolerated when used as early therapy. In PERPRISE, retention rates and seizure outcomes were also assessed in the subgroups of participants receiving PER as early (one or two prior ASMs), intermediate (three or four prior ASMs), or late (≥5 prior ASMs) adjunctive therapy. Retention rates were higher in the early (86.8%) or intermediate (80.6%) than in the late adjunctive group (62.1%), as were seizure-freedom and 50% responder rates (early vs. late use: seizure freedom, 54.2–70.8% vs. 18.2–50.0%; 50% responder, 87.5–100.0% vs. 50.0–78.9%) (30).

The retrospective, observational, multicenter COM-PER study assessed the use of PER as a first or late add-on therapy in adults with focal-onset seizures, with or without FBTCS, for 12 months (32). The retention (90.5% vs. 48.3%), seizure-freedom (71.4% vs. 13.3%), and 50% responder (85.7% vs. 28.3%) rates for total seizures were significantly higher in the first add-on group than in the late add-on group. A *post-hoc* analysis of the PERMIT study evaluated PER when used as early therapy (after failure of one or two previous ASMs) versus late add-on therapy (after failure of three or more previous ASMs) (14). At 12 months, retention was significantly higher in the early versus late add-on subgroup (67.7% vs. 62.4%), as were 50% responder rates for total seizures (76.4% vs. 47.8%), focal seizures (74.1% vs. 44.9%), and GTCS (88.1% vs. 76.7%); seizure freedom rates were significantly higher in the early versus late add-on subgroup only for total seizures (39.8% vs. 14.4%) and focal seizures (34.8% vs. 10.6%). Fewer AEs and AEs leading to discontinuation were reported in the early than in the late add-on subgroup. Differences in baseline characteristics indicated that individuals in the early add-on group were early in the disease course. Similarly, logistic regression analyses in the current study also suggest that baseline characteristics, such as the number of previous ASMs and age at epilepsy onset, might influence the response to PER.

A *post-hoc* analysis of the AMPA study evaluated the effects of PER by the number of concomitant ASMs at baseline (1, 2, or ≥ 3) (33). At 12 months, retention rates were similar between the subgroups of PWE receiving 1, 2, or ≥ 3 ASMs (56.0, 58.3, and 57.0%, respectively); however, seizure freedom rates were higher in the subgroup receiving one ASM than in the other subgroups, being 26.2, 20.2, and 13.0% in the three respective groups. Although the current study did not include a comparison between late and early add-on use of PER, it also suggests that PER should be considered early in the treatment course.

The findings of the current study are also broadly in line with the results obtained for other ASMs: both brivaracetam and lacosamide have also been shown to be effective as early add-on therapy. BRIVAFIRST was a retrospective, multicenter study of adult patients treated with adjunctive brivaracetam (34). In total, 1,029 individuals with focal epilepsy were treated with add-on brivaracetam for 12 months as early (one or two previous ASMs) or late (three or more previous ASMs) add-on therapy. The ≥50% responder rates were 60.3 and 34.3% in the early and late add-on subgroups, respectively (*p* < 0.001), and seizure freedom rates were 31.7 and 10.9%, respectively (p < 0.001). In an open-label, multicenter trial of individuals with focal-onset treated with lacosamide for 12 months as early (add-on to first ASM monotherapy) or late (add-on to one to three concomitant ASMs after at least two previous ASMs) adjunctive therapy, ≥50% responder rates were 70.3 and 50.4% in the early and late add-on subgroups, respectively, and seizure freedom rates were 37.5 and 14.9%, respectively (35). A retrospective single-center study aimed to compare the efficacy and tolerability of PER and levetiracetam when used as the first add-on therapy in patients with secondarily generalized seizures (36). Both drugs were efficacious for the treatment of seizures, and at 3, 6, and 12 months of treatment, similar efficacy outcomes in relation to seizure freedom or ≥ 75% seizure reduction were reported for the two ASMs (seizure freedom at 12 months: PER, 11/13 patients; levetiracetam, 15/17 patients) (36). However, discontinuation rates due to AEs were higher with levetiracetam than with PER, indicating that PER might have a better tolerability profile, which was likely related to the low therapeutic dose of PER used in the study (36). In line with these findings, the current study suggests that, when used as early add-on therapy, PER is efficacious at low doses (mean PER dose 4.7 mg/day), which could improve its tolerability and retention.

Study 512 provides further support for the Italian consensus statements study (15), which identified PER’s suitability as the first add-on therapy because of its unique mechanism of action and ease of use and due to evidence of a positive impact on QoL based on long-term retention data, efficacy, tolerability, no worsening of cognitive functions and sleep quality, and a low potential for drug interactions. Indeed, some studies have shown that PER does not negatively impact PWE’s quality of life (QoL) or cognition. An observational study conducted in Italy in 56 adult PWE treated with add-on PER demonstrated that it improves seizure control, does not increase levels of irritability, depression, and anxiety, and does not reduce QoL (37). The AMPA study also concluded that add-on PER does not negatively affect QoL or sleep following up to 12 months of treatment in adult patients with focal seizures, with or without FBTCS (38). A multicenter, randomized, double-blind, placebo-controlled Phase II study and its open-label extension phase assessed the effects of adjunctive PER on cognition (39). At the end of the open-label extension phase (up to 52 weeks), adjunctive PER did not have significant effects on overall cognition in adolescent patients (aged ≥12 to 18 years). The current study also suggests that PER did not have a negative impact on QoL and cognitive function; however, only a low number of patients were included in these analyses, and further studies are required.

This study has some limitations. The first limitation is that only a limited number of countries were included; thus, the cohort of PWE analyzed in the current study represents a specific socio-cultural and economic region, and results might not be applied to other populations from other geographical areas. Second, as it is common with prospective, real-world trials, there was the potential for selection bias because treatment was chosen by the treating physician for each trial participant and, as a consequence, the study population included PWE who were more likely to tolerate and/or respond to PER. Third, the lack of a comparison arm evaluating the effect of a different ASM as the first add-on therapy limits our conclusion on the relative efficacy of PER as an early add-on therapy. Finally, it should be considered that the number of patients included in some subgroups was low, limiting the power of the statistical analyses. Indeed, in some subgroups, the number of patients was too low to perform any statistical comparisons; therefore, results were analyzed only descriptively. This limitation is particularly relevant for QOLIE-10, ESS, and EpiTrack assessments.

To conclude, PER was shown to be effective across all age groups in treating a variety of seizure types and should be considered a valid option in well-defined epilepsy and when a broad-spectrum ASM is required in the management of epilepsy (for example, in cases of unclear diagnosis or multiple seizure types). Therefore, PER should be considered as an early add-on treatment following ASM monotherapy failure. PER was also generally well-tolerated, and no new safety signals emerged from the study, as suggested by the high retention rates.

## Data Availability

The raw data supporting the conclusions of this article will be made available by the authors, without undue reservation.
