# Supplementary material for: A Phase 4, multicenter, prospective, non-interventional, observational study to investigate the effectiveness and safety/tolerability of perampanel when used as first adjunctive therapy in routine clinical practice in people with epilepsy: Study 512
Source: Front Neurol. 2025 Apr 15;16:1533767. doi: 10.3389/fneur.2025.1533767 (PMC12039034; doi:10.3389/fneur.2025.1533767)

**Supplementary material**

**Supplementary Table S1. Perampanel dosing and exposure (SAS)**

|  | **Age group** | | | **Total**  **N=182** |
| --- | --- | --- | --- | --- |
| **Characteristic** | **12 to <18 years**  **n=22** | **18 to <65 years**  **n=136** | **≥65 years**  **n=24** |  |
| Modal dose, mg/day  n^a^  Mean (SD)  Median (range) | 22  6.3 (1.9)  6 (4–10) | 136  4.9 (1.9)  4 (1–12) | 24  4.6 (1.7)  4 (1–8) | 182  5.0 (2.0)  4 (1–12) |
| Daily dose, mg/day  n^a^  Mean (SD)  Median (range) | 22  6.0 (1.5)  5.7 (4–8) | 136  4.6 (1.6)  4.0 (1–10) | 24  4.1 (1.4)  3.9 (1–7) | 182  4.7 (1.7)  4.5 (1–10) |
| Daily dose, mg/day in PWE receiving carbamazepine at baseline  n^a^  Mean (SD)  Median (range) | - | - | - | 18  5.3 (1.9)  5.2 (2–10) |
| Daily dose, mg/day in PWE receiving lacosamide at baseline  n^a^  Mean (SD)  Median (range) | - | - | - | 15  3.8 (1.3)  3.9 (1–6) |
| Daily dose, mg/day in PWE receiving lamotrigine at baseline  n^a^  Mean (SD)  Median (range) | - | - | - | 24  4.4 (1.3)  3.9 (2–8) |
| Daily dose, mg/day in PWE receiving levetiracetam at baseline  n^a^  Mean (SD)  Median (range) | - | - | - | 78  4.9 (1.7)  4.5 (1–9) |
| Daily dose, mg/day in PWE receiving valproate at baseline  n^a^  Mean (SD)  Median (range) | - | - | - | 23  4.9 (1.7)  4.9 (1–8) |
| Dose titration pattern per 2 mg/day increase, n (%)  n^a^  None  Less than every week  Every 1 week  Every 2 weeks  Every 3 weeks  Every 4 weeks  More than every 4 weeks  Other | - | - | - | 182  16 (8.8)  1 (0.5)  44 (24.2)  73 (40.1)  0  17 (9.3)  4 (2.2)  25 (13.7) |
| Duration of exposure^b^, weeks  n^a^  Mean (SD)  Median (range) | 22  48.1 (13.3)  52.4 (14.7–60.3) | 136  45.4 (16.8)  52.8 (0.6–63.0) | 24  39.5 (21.0)  48.2 (30.6–48.4) | 182  44.9 (17.1)  52.2 (0.6–63.0) |
| Duration of exposure^b^ in PWE with focal onset seizures, weeks  n^a^  Mean (SD)  Median (range) | - | - | - | 137  45.0 (17.3)  52.7 (1.4–60.6) |
| Duration of exposure^b^ in PWE with GTCS in IGE, weeks  n^a^  Mean (SD)  Median (range) | - | - | - | 36  45.6 (16.1)  52.4 (0.6–63.0) |
| Duration of exposure^b^ in PWE receiving carbamazepine at baseline, weeks  n^a^  Mean (SD)  Median (range) | - | - | - | 18  49.1 (14.7)  54.1 (5.0–60.6) |
| Duration of exposure^b^ in PWE receiving lacosamide at baseline, weeks  n^a^  Mean (SD)  Median (range) | - | - | - | 15  29.8 (22.2)  37.3 (1.4–57.1) |
| Duration of exposure^b^ in PWE receiving lamotrigine at baseline, weeks  n^a^  Mean (SD)  Median (range) | - | - | - | 24  46.0 (15.8)  52.3 (5.4–60.3) |
| Duration of exposure^b^ in PWE receiving levetiracetam at baseline, weeks  n^a^  Mean (SD)  Median (range) | - | - | - | 78  46.0 (16.3)  52.8 (0.9–63.0) |
| Duration of exposure^b^ in PWE receiving valproic acid at baseline, weeks  n^a^  Mean (SD)  Median (range) | - | - | - | 23  47.4 (15.1)  52.0 (0.6–60.0) |
| Duration of exposure^b^ in PWE receiving EIASM^c^, weeks  n^a^  Mean (SD)  Median (range) |  |  |  | 34  46.0 (17.4)  53.6 (3.7–60.6) |
| Duration of exposure^b^ in PWE receiving non-EIASM^c^, weeks  n^a^  Mean (SD)  Median (range) |  |  |  | 148  44.7 (17.0)  52.0 (0.6–63.0) |

^a^Number of PWE for whom datum was available; ^b^Duration of exposure = date of last dose of study drug – date of first dose of study drug + 1. ^c^EIASMs, carbamazepine, eslicarbazepine acetate, oxcarbazepine, and phenytoin; non-EIASMs, levetiracetam, lamotrigine, valproate, lacosamide, topiramate, clonazepam, zonisamide, and brivaracetam. ASM, antiseizure medication; EIASM, emzyme-inducing antiseizure medication; GTCS, generalized tonic-clonic seizure; PWE, people with epilepsy; SAS, Safety Analysis Set; SD, standard deviation;

**Supplementary Table S2.** Summary of univariate and multivariate logistic regression analyses of predictors of (A) Retention on PER over 12 months (SAS), (B) Pragmatic seizure freedom at 12 months (FAS) and (C) Completer seizure freedom (FAS)

| **(A) Retention rate at 12 months** | | | |
| --- | --- | --- | --- |
| **Variable** | **n** | **OR (95%CI)** | **p-value** |
| **Multivariate analysis (n=178)** | | | |
| Baseline seizure frequency |  | 1.007 (0.988, 1.027) | NS |
| Number of prior ASM monotherapy  1  ≥2 |  | 0.782 (0.339, 1.803) | NS |
| Type of concomitant ASM  EIASM  Non-EIASM |  | 0.598 (0.218, 1.638) | NS |
| Age at diagnosis |  | 0.973 (0.952, 0.993) | **0.0096** |
| Time since diagnosis |  | 0.993 (0.952, 1.037) | NS |
| Etiology^a^  Genetic  Others |  | 0.855 (0.193, 3.798) | NS |
| Etiology^a^  Structural  Other |  | 2.046 (0.771, 5.430) | NS |
| Etiology^a^  All other etiologies  Other |  | 0.586 (0.142, 2.419) | NS |
| Previous psychiatric history  No  Yes |  | 1.585 (0.577, 4.354) | NS |
| Seizure type  Focal-onset seizures  Primary generalized seizures |  | 0.734 (0.228, 2.364) | NS |
| **(B) Pragmatic seizure freedom** | | | |
| **Variable** | **n** | **OR (95%CI)** | **p-value** |
| **Univariate analysis** |  |  |  |
| Baseline seizure frequency | 174 | 0.99 (0.98, 1.01) | NS |
| Number of prior ASM monotherapy  1  ≥2 | 106  67 | 0.39 (0.20, 0.78) | **0.072** |
| Seizure type  Focal-onset seizures  GTCS | 132  33 | 1.61 (0.74, 3.49) | NS |
| Type of concomitant ASM  EIASM  Non-EIASM | 141  33 | 0.72 (0.32, 1.63) | NS |
| Age at diagnosis | 172 | 1.01 (1.00, 1.03) | NS |
| Time since diagnosis | 172 | 0.97 (0.94, 1.01) | NS |
| Etiology^a^  Genetic  Others | 18  54 | 0.63 (0.20, 1.91) | NS |
| Etiology^a^  Structural  Other | 43  29 | 1.30 (0.50, 3.39) | NS |
| Etiology^a^  All other etiologies  Other | 11  61 | 1.20 (0.33, 4.37) | NS |
| Previous psychiatric history  No  Yes | 141  33 | 0.86 (0.38, 1.91) | NS |
| **Multivariate analysis (n=178)** |  |  |  |
| Baseline seizure frequency |  | 0.992 (0.997, 1.008) | NS |
| Number of prior ASM monotherapy  1  ≥2 |  | 0.405 (0.185, 0.884) | **0.0232** |
| Type of concomitant ASM  EIASM  Non-EIASM |  | 0.499 (0.184, 1.357) | NS |
| Age at diagnosis |  | 1.0 (0.981, 1.020) | NS |
| Time since diagnosis |  | 0.983 (0.940, 1.027) | NS |
| Etiology^a^  Genetic  Others |  | 0.537 (0.140, 2.058) | NS |
| Etiology^a^  Structural  Other |  | 2.256 (0.981, 5.189) | NS |
| Etiology^a^  All other etiologies  Other |  | 1.024 (0.246, 4.265) | NS |
| Previous psychiatric history  No  Yes |  | 0.842 (0.349, 2.032) | NS |
| Seizure type  Focal-onset seizures  Primary generalized seizures |  | 2.129 (0.753, 6.022) | NS |
| **(C) Completers seizure freedom** | | | |
| **Variable** | n | **OR (95%CI)** | **p-value** |
| **Univariate analysis** |  |  |  |
| Baseline seizure frequency | 135 | 0.991 (0.979, 1.004) | NS |
| Number of prior ASM monotherapy  1  ≥2 | 84  51 | 0.360 (0.173, 0.748) | **0.0062** |
| Type of concomitant ASM  EIASM  Non-EIASM | 25  110 | 0.717 (0.296, 1.734) | NS |
| Age at diagnosis | 133 | 1.021 (1.002, 1.039) | **0.0277** |
| Time since diagnosis | 133 | 0.969 (0.935, 1.005) | NS |
| Etiology^a^  Genetic  Others | 15  120 | 0.737 (0.247, 2.199) | NS |
| Etiology^a^  Structural  Other | 35  100 | 1.511 (0.697, 3.275) | NS |
| Etiology^a^  All other etiologies  Other | 8  127 | 1.983 (0.454, 8.652) | NS |
| Previous psychiatric history  No  Yes | 108  27 | 0.740 (0.315, 1.742) | NS |
| Seizure type  Focal-onset seizures  Primary generalized seizures |  | 1.583 (0.674, 3.719) | NS |
| **Multivariate analysis (n=133)** |  |  |  |
| Baseline seizure frequency |  | 0.990 (0.974, 1.006) | NS |
| Number of prior ASM monotherapy  1  ≥2 |  | 0.367 (0.150, 0.899) | **0.0284** |
| Type of concomitant ASM  EIASM  Non-EIASM |  | 0.566 (0.190, 1.683) | NS |
| Age at diagnosis |  | 1.014 (0.991, 1.037) | NS |
| Time since diagnosis |  | 0.998 (0.949, 1.049) | NS |
| Etiology^a^  Genetic  Others |  | 0.586 (0.137, 2.501) | NS |
| Etiology^a^  Structural  Other |  | 2.048 (0.811, 5.172) | NS |
| Etiology^a^  All other etiologies  Other |  | 1.556 (0.264, 9.160) | NS |
| Previous psychiatric history  No  Yes |  | 0.766 (0.294, 1.996) | NS |
| Seizure type  Focal-onset seizures  Primary generalized seizures |  | 2.693 (0.817, 8.876) | NS |

^a^Genetic etiology: defined genetic cause; structural etiology: CNS infection, stroke, structural brain anomalies or malformations or vascular brain anomalies

ASM, antiseizure medication; CI, confidence interval; EIASM, emzyme-inducing antiseizure medication; FAS, Full Analysis Set; OR, odds ratio; GTCS, generalized tonic-clonic seizures

**Supplementary Table S3.** Responder rate and worsening seizure rate by detailed seizure types at 6 and 12 months

| **Responder rate** |  |  |
| --- | --- | --- |
|  | **6 Months*** | **12 Months*** |
| Focal-onset seizure, n/N (%)  Focal aware motor onset  Focal aware non-motor onset  Focal impaired awareness  FBTCS | 11/17 (64.7)  13/20 (65.0)  20/44 (45.5)  28/40 (70.0) | 12/17 (70.6)  13/20 (65.0)  24/44 (54.5)  31/40 (77.5) |
| Generalized-onset, n/N (%)  Absence  Atypical  Myoclonic  Tonic  Atonic  Tonic-Atonic  Clonic  Tonic-clonic  Epilepsy spasm  Other | 7/12 (58.3)  0  8/11 (72.7)  2/3 (66.7)  0  0  1/1 (100)  16/23 (69.6)  0  0 | 8/12 (66.7)  0  10/11 (90.9)  2/3 (66.7)  0  0  1/1 (100)  15/23 (65.2)  0  0 |
| **Seizure worsening rate** |  |  |
|  | **6 Months*** | **12 Months*** |
| Focal-onset seizure, n/N (%)  Focal aware motor onset  Focal aware non-motor onset  Focal impaired awareness  FBTCS | 3/17 (17.6)  2/20 (10.0)  7/44 (15.9)  3/40 (7.5) | 3/17 (17.6)  1/20 (5.0)  5/44 (11.4)  4/40 (10.0) |
| Generalized-onset, n/N (%)  Absence  Atypical  Myoclonic  Tonic  Atonic  Tonic-Atonic  Clonic  Tonic-clonic  Epilepsy spasm  Other | 1/12 (8.3)  0  0/11  0/3  0  0  0/1  4/23 (17.4)  0  0 | 0/12  0  0/11  0/3  0  0  0/1  4/23 (17.4)  0  0 |

*Subjects who withdrew prior to this period or had less than 4 weeks of data during this period had their last 3 months of treatment carried forward.FBTCS, focal to bilateral tonic-clonic seizures

**Figure S1.** **Changes from baseline in seizure frequency at 6 months (measured over months 4‒6) and 12 months (measured over months 7‒12) in (A) the overall population and the subgroups of PWE aged 12 to <18, ≥18 to <65, and ≥65 years, (B) focal seizures, and (C) generalized seizures (FAS).** FAS, Full Analysis Set; FBTCS, focal to bilateral tonic-clonic seizures; PWE, people with epilepsy


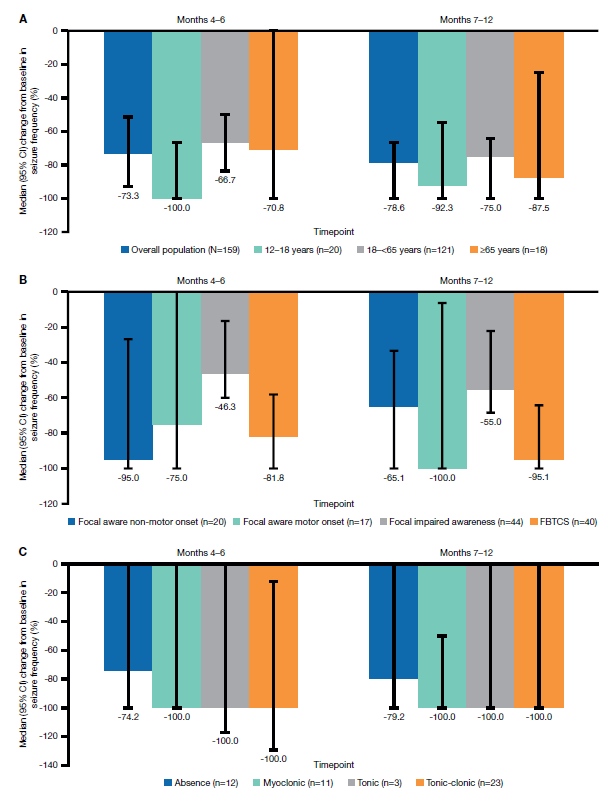


**Figure S2.** R**esponder rate, seizure freedom rate, and seizure worsening rate in overall population and subgroups of PWE aged 12 to <18, ≥18 to <65, and ≥65 years at (A) 6 months and (B) 12 months (FAS).** FAS, Full Analysis Set; PWE, people with epilepsy


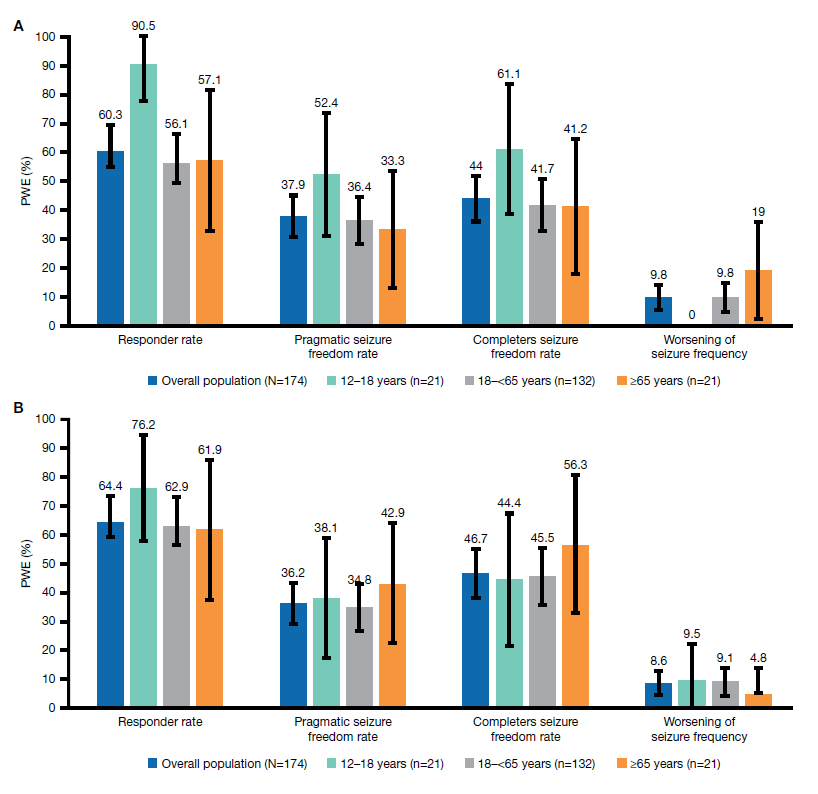

Supplement: Supplementary file 1 [file Supplementary_file_1.docx]
